# Exploration of the Knee Joint

**Published:** 1899-08-05

**Authors:** 


### EXPLORATION OF THE KNEE JOINT.

The main joints in the lower extremity differ from those in the upper limb in this among other respects that, unless they are in sufficiently perfect repair to enable them to bear the whole weight of the body, they are practically of but little service to their owner. Greatly as the utility of the upper extremity is interfered with by anything which prevents the free mobility of its joints, by a little humouring the limb can often be made to do fairly good service even although it may be lame. But in the lower limb there is no such adaptability, and if the knee, for example, cannot bear the whole weight of the body freely and without pain, and if it cannot perform its proper functions with the whole weight of the body pressing upon it, it might as well not exist. Thus not only is the knee one of the most important of all the joints, but it is one in regard to the diseases of which nothing short of a perfect cure is of any material service to the patient. Hence the supreme importance of an accurate diagnosis of the exact condition which is the cause of what, so far as its physical signs are concerned, may seem to be only a simple synovitis.

Mr. Walsham, in an address recently delivered, says that whether we have to do with loose bodies in the joint, detachment or displacement of the semilunar cartilages, enlargement with nipping of hypertrophied synovial fringes, or elongation of the ligamentum patellæ, we may find certain somewhat similar symptoms, namely, effusion into the synovial membrane, a feeling of weakness or disability in the joint, some limitation to the range of flexion and extension, pain—often sudden and even excruciating during certain movements—and at times a sensation as of something slipping in or round the joint. But in all these conditions the most obvious sign is apt to be synovial effusion, and thus the case is likely to be diagnosed as one of synovitis. Such a diagnosis is correct, but it does not go far enough. "It is synovitis and something more, and unless this additional something is made out, except it consists in a mere rupture of some ligamentous fibres, the routine treatment of synovitis will not cure the patient." In such cases then it may become necessary to open the knee joint if a radical cure is to be obtained; and in considering the propriety of undertaking such an operation in cases in which it seems to be demanded, it is worth while to remember how alike the peritoneum and the synovial membranes are to each other, both in structure and in physiological functions, for it no doubt is the case that they will not only react in a similar manner when subjected to injury, but will be found to possess the same power of physiological repair. "When proper precautions are taken," says Mr. Walsham, "a healthy synovial membrane may be treated as fearlessly as may the peritoneum, and with as great a certainty of success." Among the essentials to success may be enumerated the following points: (1) The preparation of the patient, including asepticising the skin, accustoming the patient to the use of the splint, and removing any effusion that may be present in the joint; (2) the arrest of all hæmorrhage before the capsule is opened, and the thorough cleansing of the joint afterwards; (3) accurate suture of synovial membrane and capsule; (4) absolute rest till the skin wound is soundly healed; and (5) early passive movements and massage.

It may be well here to mention the rules which are in use in Mr. Walsham's wards at St. Bartholomew's Hospital for asepticising the skin for every operation both great and small, for it is only by carefully observing a definite plan of treatment that anything like uniformity of results can be relied upon: (1) Shave the surface to be prepared (if necessary). (2) Scrub well with soap and water, and rub in ether. (3) Wash your hands, and sterilise them in biniodide of mercury lotion (1 in 1,000). (4) Wash surface with biniodide of mercury lotion (1 in 5,000) for two minutes. (5) Wash this off with biniodide of mercury lotion (1 in 2,000). (6) Apply, first, sterilised carbolic gauze wrung out in biniodide of mercury lotion (1 in 4,000); second, protective india-rubber tissue wrung out in carbolic acid (1 in 20); third, absorbent or boracic wool; and fourth, a bandage.

